# Stepping Up and Stepping In: Exploring the Role of Nurses in Supporting Grandparents Raising Grandchildren

**DOI:** 10.1177/10748407221124854

**Published:** 2022-09-28

**Authors:** Christina Murray, Laura Bain, Patrice Drake, Don Avery

**Affiliations:** 1 University of Prince Edward Island, Charlottetown, Canada; 2 Building GRAND-Families, Bonshaw, Prince Edward Island, Canada

**Keywords:** grandparents raising grandchildren, narrative inquiry, patient and family-centered care, family nursing

## Abstract

This study focused on the experiences of grandparents raising grandchildren in rural, Prince Edward Island, Canada. Termed grand-families, there are numerous reasons why grandparents must step up and step in to care for their grandchildren. Often these reasons are related to their adult children’s struggles with mental illness and substance use disorders. Adopting Clandinin and Connelly’s approach to narrative inquiry, we present findings from the conversational interviews conducted with 12 grandparents raising their grandchildren. Interview data were analyzed through the narrative dimensions of time, place, and relationship. Findings are presented as rich narratives which illuminate the evolution and storied experiences of grand-families. Particularly revealing are the challenges grandparents face as they navigate various systems, including health care, that do not acknowledge the uniqueness of their family form. Nurses work with grand-families across varied clinical settings. Grounded within the philosophy of Patient and Family Centered Care and family nursing theory, this article offers recommendations for supportive interventions that nurses can implement when caring for grand-families across clinical settings. This study has the potential to facilitate the development of evidence-based supports and services, which are responsive to the needs, realities, and complexities of grand-families.

Grandparents raising their grandchildren is a worldwide phenomenon that crosses cultural and socioeconomic spheres (Chan et al., 2019; Hadfield, 2014; McLaughlin et al., 2017). In Canada, the departure from the archetypal concept of the nuclear family is evident with the most recent census data reporting more than half a million (553,855) children aged 0 to 14 living in multigenerational homes with at least one of their grandparents (Statistics Canada, 2022). Of these families, 36,860 children live without their parents, and their grandparents are the primary care providers (Statistics Canada, 2022).

We have adopted the term *grand-family* to define a family that is formed due to extenuating factors resulting in grandparents simultaneously functioning in the roles of a grandparent as well as acting as a primary parent to one or more grandchildren who live with the grandparents full-time. Grand-families are established for many reasons including parental mental illness, substance use disorders, incarceration, long-distance employment separation, young maternal age, physical disability, and death (Avery & Novoa, 2022; Choi et al., 2016; Lefebvre & Rasner, 2017; Martin et al., 2021; Murray et al., 2022; Van Etten & Gautam, 2012). Grand-families are associated with positive outcomes for the grandchild; however, studies have shown an increased risk of negative psychological and physical outcomes for the grandparents (Chan et al., 2019; Hadfield, 2014; Zuchowski et al., 2019).

The traditional North American view is that the parent of the child will provide full-time care and raise their child. When a grand-family is formed, the duration in which a grandparent must provide care to their grandchild is often unknown and in flux. Through our research, we conceptualized and developed the continuum of grand-family evolution to reflect the dynamic nature of grand-families (Figure 1). Grand-families may be formed on a temporary, short-term basis, after which the child returns to their parents on a full-time basis, for example, if a parent is working away from home or experiencing an inpatient medical stay. Grand-families may need to be formed for a more extended and/or indefinite period of time such as when a parent is living with an active substance use addiction or is incarcerated. Finally, grand-families may need to be permanently formed when their grandchild’s parent(s) have lost custody of the child or have died.

**Figure 1.**
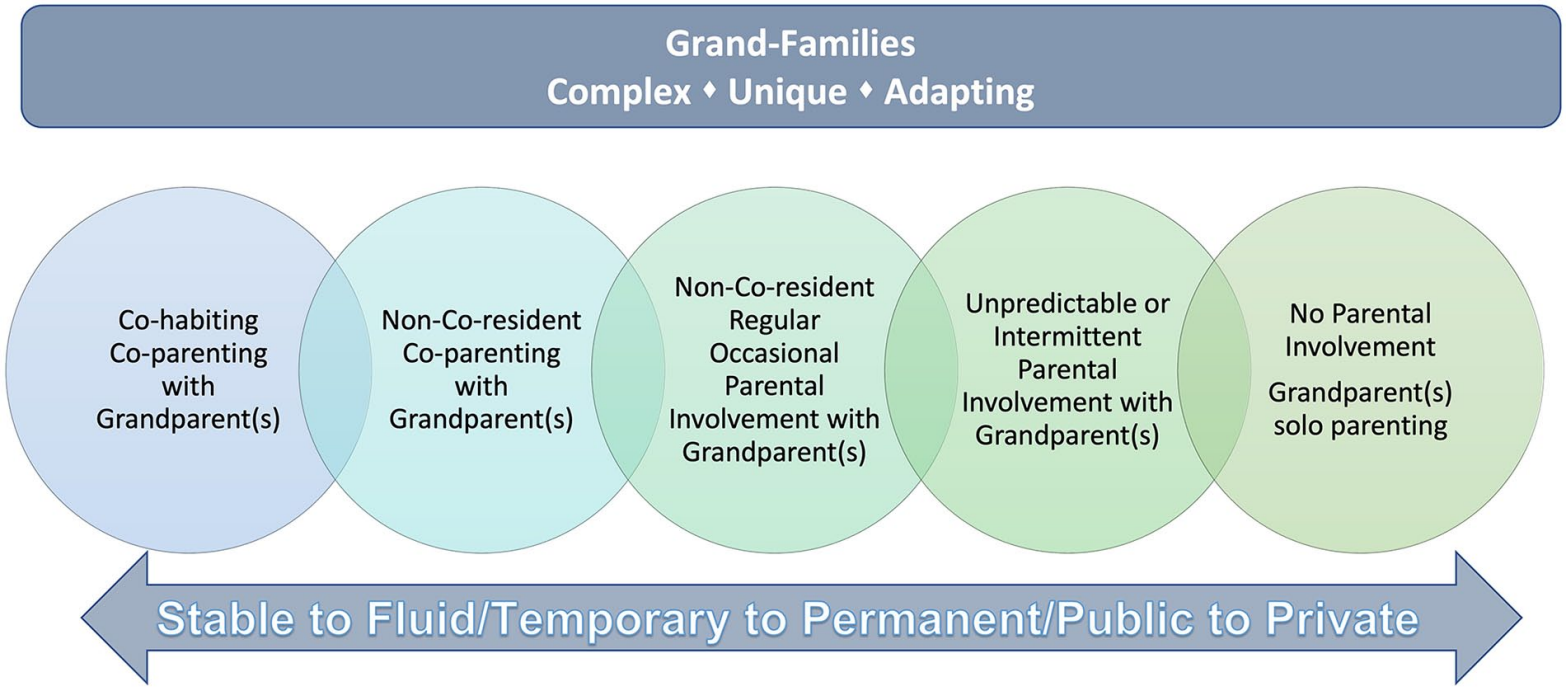
Continuum of grand-family evolution.

Many grand-families do not have formal legal guardianship agreement. The reasons for this include grandparent fears about being assessed by a social worker and being viewed as an unfit guardian and as such, having their grandchild being lost to the foster system; high legal costs related to applying for formal guardianship; and estrangement from their adult children (Avery & Novoa, 2022; Choi et al., 2016; Hadfield, 2014; Lewis & Seponski, 2012; Lewis et al., 2018; Zuchowski et al., 2019). The lack of a formal guardianship agreement for a grandchild is problematic because the legal authority for decision-making remains with the parent who is no longer participating/acting in the role of primary caregiver to their child. This leads to challenges for grandparents as they may be the full-time caretaker to their grandchildren yet do not have the legal right to consent to care decisions that may be required for the child such as in the delivery of health care services (Avery & Novoa, 2022; Choi et al., 2016; Hadfield, 2014; Lewis & Seponski, 2012; Lewis et al., 2018; Zuchowski et al., 2019).

Grandparents may struggle to support their grandchild who have experienced past trauma while in the care of their biological parent. In addition, there may be deterioration and/or estrangement in their relationships with their adult children as a result of the circumstances that have contributed to the formation of the grand-family. As grandparents transition into grand-families, they may experience feelings of grief and loss related to their perceptions of what grandparenthood would be versus their lived realities. This may result in increased stress, and pressure as they navigate the role of being both grandparent and primary parent to their grandchildren (Avery & Novoa, 2022; Backhouse & Graham, 2013; Choi et al., 2016; Lefebvre & Rasner, 2017; Martin et al., 2021; Murray et al., 2022).

Intergenerational family members face individual challenges that permeate across the grand-family. Grandparents leading grand-families may also be managing chronic illnesses, complex health issues, and other health comorbidities related to advanced age (Martin et al., 2021). This has the potential to impact their quality of life, life expectancy, financial stability, and may also contribute to additional uncertainties about the future for grand-families (Avery & Novoa, 2022; Choi et al., 2016; Martin et al., 2021; Milan et al., 2015). Adult children struggling with substance use disorders have the potential to cause disruption and disarray within grand-families due to the unpredictable nature of the illness. Grandchildren may live with the physical, cognitive, and developmental impacts of being born to a parent living with a substance use disorder. Grandchildren may also live with mental and emotional health challenges such as anxiety, depression, post-traumatic stress disorder, attachment disorder, and separation anxiety as a result of the experiences they had with their parents prior to coming to stay with their grandparents. While these challenges are experienced individually by family members, one cannot overlook the impact on the grand-family as a whole.

In this article, we will present an overview of the experiences of grandparents raising grandchildren in rural Prince Edward Island, Canada. We will begin with a brief review of current literature followed by sharing key findings and insights related to the semi-structured interviews from our *It Takes a Village to Raise a Grandchild* project. Particular focus will be provided on how a lack of understanding about grand-families by various professionals, including nurses, has had a detrimental impact on family well-being. We will present the lived experience of grandparents raising grandchildren, and what grandparents wished nurses knew about them. Grounded within philosophy of Patient and Family-Centered Care and Family Nursing, this article will conclude with recommendations for supportive interventions that nurses can implement when caring for grand-families across clinical settings.

## Literature Review

A literature review was conducted to examine the scope of evidence that currently exists regarding grand-families. Challenges arose given the varied terminology used to describe the phenomenon of grandparents raising grandchildren. The most common term used is “grandfamily” with various spellings: “grand-family” and “grandfamily” (Dolbin-MacNab & O’Connell, 2021; Fruhauf et al., 2022). Other terms include “kinship care,” “skipped generation homes” (Dolbin-MacNab & O’Connell, 2021; Statistics Canada, 2017), “multigenerational homes” (Fruhauf et al., 2022); “Grandparents raising” or “Grandparents raising grandchildren” (Dolbin-MacNab & O’Connell, 2021; Kelley et al., 2001; Lee et al., 2018; Shaklee et al., 2012); “Grandparent Headed Families” or “Grandparent Headed Households” (Lee et al., 2018); and “Custodial Grandparents” (Fruhauf et al., 2022). “Kinship Care,” while applicable for literature searches, is not specific enough to fit our chosen population of grandparents (Dolbin-MacNab & O’Connell, 2021). Statistics Canada (2017) used the term “skip-generation family” and defined it as a census family of grandparents and grandchildren without the presence of parents in the home. However, we believe this term is still too general and could be used to describe a great aunt or uncle raising a grandchild. Our chosen term is “grand-family,” as it is specific to our population of interest: grandparents. The decision was made by the research team to adopt the term grand *hyphen* family to distinctly signify how these families are impressive and deserving of special acknowledgment. Grand-family also serves to highlight the “grandness” of this family form as we believe in a strengths-based approach and empowerment of grandparents raising grandchildren.

The articles reviewed were diverse and reflected global perspectives on the experiences of grand-families with sources originating in Australia, China, Europe, Africa, Canada, and the United States. We prioritized meta-analyses and systematic reviews and those relating to health care (Chan et al., 2019; Hadfield, 2014; Kelley et al., 2001; McLaughlin et al., 2017). There was convincing evidence that grandparents raising grandchildren experienced an increased risk to their physical, physiological, and mental health, particularly depression, as compared with grandparents who were not primary caregivers for their grandchildren (Chan et al., 2019; Hadfield, 2014; Kelley et al., 2001; McLaughlin et al., 2017). Grandparents intentionally, but often with little preparation, take on the role of heading a grand-family at a time of crisis, and this choice is to protect the grandchild from an alternative placement (Hadfield, 2014). There is a lack of research about minority groups of grandparents raising grandchildren including grandfathers raising grandchildren, Indigenous caregivers, and grand-families in rural areas (Hadfield, 2014).

Chan et al. (2019) highlighted the global context of grand- families and then evaluated the effectiveness of interventions with respect to a wide spectrum of well-being outcomes including mental health, physical health, general health, empowerment/life satisfaction, family support, and family relationship. The authors concluded that programs using supportive and educational components resulted in significant increases in social support and parenting skills as well as significant decreases in the grandchildren’s behavioral problems (Chan et al., 2019). Based on evidence from different evaluation studies, there is no one-size-fits-all program; however, interventions work best when taking a family-based approach and when the needs of grandparents, parents, and grandchildren are considered (Chan et al., 2019; Hadfield, 2014).

A systematic review conducted by McLaughlin et al. (2017) on the effectiveness of interventions for grandparent caregivers, critically appraised 21 studies from the United States, Australia, and Africa. The review supports the findings from Chan et al. (2019) that interventions are beneficial and the authors further illuminated that those interventions within the cognitive-behavioral field have the greatest empirical evidence support. The authors confirm that the reasons behind the formation of grand-families are complex and often involve traumatic events that lead to a crisis intervention by the grandparents (McLaughlin et al., 2017). This crisis impacts the psychological and physical health of grandparent caregivers which is unsurprising, given the stress and trauma, and is compounded by socioeconomic and psychosocial factors (McLaughlin et al., 2017). While this quantitative body of knowledge is important, the grandparent’s story as they would tell it, is missing and is necessary to illuminate their struggles and triumphs as they care for their grandchildren.

Our review of the literature revealed a gap in the breadth and depth of qualitative research related specifically to the lived experiences of grandparents raising grandchildren. For example, Kelly et al. (2001) excluded all qualitative studies from their meta-analysis and only included studies from the United States. McLaughlin et al., (2017) only had four qualitative studies represented in a systematic review of 21 studies. While research reflected the phenomenon of grand-families across multiple countries, the majority of the research reviewed was conducted in the United States and in urban settings. Many of the recommendations called for future grand-family research to address the gap in rural communities. Minimal research on grand-families exists in Canada and, from our review, nothing has been published specific to nurses. While we discovered one study specific to pediatricians working with grand-families in Nova Scotia Canada, there exists a gap in regard to nursing care of grand- families (Martin et al., 2021).

The lack of qualitative articles and lack of publications that reflect the rural experience and the role of nursing working with grand-families highlights the need for more understanding of grand-families experience raising grandchildren in their own words. This information is necessary to implement effective interventions at the health, social, justice, and education levels. Researchers agree that grand-families face multidimensional, complex problems with substantial variation in the caregiving experience (Chan et al., 2019; McLaughlin et al., 2017). There remains a lack of awareness regarding the of issues facing grand-families from community engagement systems including health care. The *It Takes a Village to Raise a Grandchild* study offers a new contribution to our understanding regarding the evolution and adaptability of grand-families in Canada.

## Method

Recognizing the lack of current qualitative research focused on the experiences of Canadian grand-families and health care practitioners, we embarked on a 2-year, multimethods qualitative research study titled: *It Takes a Village to Raise a Grandchild.* Members of the project team included nurses with decades of direct clinical practice and research experience working with families; a great-grandfather who has lived experience leading a grand-family; and is a community champion advocating for the rights of grand-families in Canada; and the Chief Executive Officer of a national Canadian institute whose purpose is to advance family issues. Fundamental to the conceptualization of this project was the intentional development of partnerships and active engagement between members of the research team and with each participant in our study. In developing research relationships, we were cognizant of Cargo and Mercer’s (2008) recommendations that

Equal participation of academic and non-academic partners is the ideal for many participatory research approaches to help partnerships balance scientific excellence with social and cultural relevance; foster ownership, capacity building, and empowerment of nonacademic partners; and translate research knowledge into action. (p. 332)

### Study Design

The goal of this study was to raise awareness and increase understanding about the uniqueness of grand-families. Our objectives were to

1. illuminate the lived experiences of grandparents leading grand-families;
2. facilitate opportunities for grandparent and professional collaboration to uncover issues impacting grand-families; and
3. identify family-centered interventions.

In this multiphased, multimethods study, three distinct approaches to data collection were adopted: (a) conversational semi-structured interviews with grandparents leading grand-families; (b) focus group interviews with grandparents leading grand-families; and (c) a collaborative community workshop. These approaches were chosen to raise awareness, deepen understanding, and elicit multiple perspectives regarding the experiences of grandparents who are leading grand-families. Each method was conducted independently using distinct data collection instruments. The focus of this article is on discoveries made from our individual conversational interviews. Due to the scope of this article, we have selected to present only the findings from these interviews as these showcased the uniqueness of grand-families as a distinct family form, the circumstances that led to them becoming a grand-family, their present realities of being a grand- family, as well as their future hopes and aspirations. These interviews also illuminated the challenges faced by grand-families as they navigated systems and practitioners that they interact with regularly. As such, we believe that sharing the findings from this piece as part of our larger study offers a valuable contribution for the nursing of these families.

### Research Methodology

Clandinin and Connelly’s (2000) approach to narrative inquiry was the research methodology used to guide the semi-structured interviews. Narrative inquiry is a way of understanding and representing experience and, as narrative inquirers, we understand narrative as both the phenomenon under study and the methodology behind understanding experience. Grounded within John Dewey’s (1938, 1998) philosophical assumptions regarding experience, narrative inquirers are situated within a three-dimensional space of continuity—past, present, future interaction and relationship with others, and place. Central to the epistemological foundation of narrative inquiry is an emphasis on the development and nurturing of research relationships between a researcher and a participant (Clandinin & Connelly, 2000).

Clandinin and Connelly’s (2000) form of narrative research aligned particularly well with our desire to engage with research participants and understand the experience of grand-families more fully. Researchers embarking in narrative research have a keen desire to collaborate with participants and help raise awareness about phenomenon in a way that nurtures trust and flattens actual or perceived hierarchies. This is achieved through working with research participants in a co-participatory manner that invites a sharing of lived experiences in a storied way using a variety of data collection methods (Clandinin & Connelly, 2000).

### Interview Protocol

To gain a more comprehensive understanding of the storied lives of grandparents leading a grand-family, we conducted individual, semi-structured conversational interviews with 12 grandparents who are raising their grandchildren. We chose a conversational approach to interviewing, as it aligned well with our theoretical underpinnings and desire to promote participant engagement. According to Clandinin and Connelly (2000), conversational interviews are built on mutual respect, trust, and mutual sharing of stories and experiences between the participants and researchers. Questions were designed to encourage fluidity, flexibility, and openness and reflected Clandinin and Connelly’s narrative dimensions.

During conversational interviews, grandparents were invited to share their experiences and to reflect on their time as a grandparent prior to becoming a grand-family, to share their present experiences leading a grand-family, and their future hopes and dreams for their families. Reflecting on the past, we discussed the experiences of role transition from grandparent to a grandparent raising grandchildren, and the formation of a grand-family. Focusing on the present, we explored the day-to-day life experiences of grandparents as they function in the duality of grandparent and primary parent to children. The final overarching question explored the hopes and fears grandparents had as they reflected on their futures and that of their grand-families.

### Sampling Procedure and Participants

A purposive and snowballing approach to participant recruitment was used for this study. Information about the study was shared with grandparents who attended grandparents raising grandchildren support group meetings, posters placed throughout various places grandparents frequented, and through media interviews that had been conducted about this study with the Principal Investigator. Inclusion criteria for participation in this study required grandparents to be

leading a grand-family for 2 or more years;
caring for at least one grandchild on a full-time basis for a minimum of 2 years;
English speaking; and
willing to share their stories of experience leading a grand-family.

The rationale for selecting 2 years as the time period required for participation in the study was based on our desire to work with participants in a respectful manner, reflective of trauma-informed care. By establishing this time period as an inclusion criterion, we were cognizant of the often-traumatic circumstances that grandparents and children have experienced leading up to the point of them needing to become a grand-family. As a research team, we recognized that there was a period of adaption and adjustments required during the immediate time of transition when grandparents and grandchildren adapted to living together as a family unit. In the planning of this study, grandparents shared that they felt that it took about 2 years for their grand-family to feel like they had some stability and clarity in their roles and responsibilities. This does not imply that a grand-family is formed after 2 years of consecutive care.

Twelve grandparents participated in our conversational interviews. Grandparents ranged in age from 44 to 83 and, at the time of the interviews, were providing full-time care for between one and four children. One grandfather participated in our study, with the remaining 11 being grandmothers. Five grandparents were still working in paid full-time employment. Interviews were conducted at a mutually agreed upon location and ranged between 45 min and 1.5 hr. Prior to beginning our conversational interviews, a study information letter was shared and written consent to participate was obtained from each participant. Due to the potentially sensitive stories of experiences that could be shared by participants, a decision was made to have all interviews conducted by the study’s Principal Investigator who was a Registered Nurse with more than 20 years of experience in family nursing and supporting families who have lived through traumatic experiences.

Prior to conducting our conversational interview, ethical approval for this study was received from the University of Prince Edward Island Research Ethics Board. Identifying data related to this study is stored in a locked filing cabinet at the lead author’s university and will be kept for a period of 5 years.

### Data Analysis

Individual conversational interviews were digitally recorded and transcribed verbatim. A thematic approach to data analysis then occurred and aligned within Clandinin and Connelly’s (2000) narrative dimensions of place, time, and relationship. The research team engaged in an iterative process of independently reviewing and coding interview transcripts and documenting emerging themes. Data saturation occurred when a redundancy of recurrent themes and anticipation of recurring themes was expected and evident in the subsequent interview transcripts. This informational redundancy occurred after the review of Interview 8; however, due to the richness of interview data and depth of sharing among participants during conversational interviewing, a decision was made to thoroughly analyze all 12 interview transcripts.

The research team then came together and discussed their individual analyses. Consensus agreement was achieved with the identification of predominant themes, and no major discrepancies were identified. In addition, field notes and a reflexive journal documenting key insights and reflections throughout the study were kept. Upon review of our identified themes, field notes, and reflexive journal, we were struck by how many grandparents did not feel supported by professionals that they regularly engaged with. To understand this more fully we returned to our participants and asked them to share more about what they wished nurses knew about their grand-families. Grandparents shared their reflections in writing which were then transcribed verbatim and analyzed by our team where additional themes were identified.

## Findings

Data analysis of the grandparent’s stories revealed rich narratives that illuminated the experiences of grandparents caring for grandchildren as the primary parent/caregiver. The following discussion will describe the predominant themes that were revealed and will present them as *being, considering the future, and supporting grand-families.*

### Being: “There Was No Being A Grandparent. It Was Bang, We’re Parents Again”

The analysis of our conversational interviews revealed that becoming a grand-family was often sudden and in response to a traumatic life event that resulted in their grandchild needing protection, connection, and a safe home. All grandparents referred to a pivotal moment or sudden crisis within their family and the lives of their adult children that led to them needing to step up and step in to care for the grandchildren and become a grand-family. Often these crises were related to drugs, alcohol, mental illness, and/or being arrested due to criminal activities. A grandparent shared, “there was no, really being a grandparent. It was bang, we’re parents again.” Another grandmother explained,

My husband’s cell phone and my cell phone both rang at the same time. It was simultaneously. It was both at the same time. I had child protection. He had the RCMP [Royal Canadian Mounted Police]. “You need to get down here. Come and take the baby.” We did. He’s been with us ever since.

Many grandparents spoke at length about how unprepared they were to become a grand-family. No one imagined that at their age and stage in life that they would be parenting children again. However, when they were asked to take care of their grandchildren, they did so without question nor consideration about how this decision would permeate all aspects of their lives; from their careers, personal finances, relationships with other family members and friends, future plans and in particular their preconceived vision of what it would be like to be a grandparent. The sudden need to transition and adapt their lives for the well-being of their families was striking. As one grandparent explained,

At the beginning, it was really, really hard. Being a grandparent raising grandchildren because I already raised my own. And I wasn’t quite sure what this was going to be like, but I knew that I couldn’t allow them to go into foster care or anything else. I had a job on Thursday, and I had no job on Friday, because I had the kids.

Eleven of the 12 grandparents had adult children who were living with mental illness and/or substance abuse disorders resulting in them being in a place where they were unable to provide care for their child(ren). This created chaos and a feeling of constant uncertainty within the family unit as grandparents and grandchildren did not know when their loved one would be well enough to engage with the family. Grandparents struggled to explain to their grandchildren why their parent was unable to look after them. Explanations from grandparents varied depending on the child’s developmental age and past experiences living with their parents. As one participant whose child was living with a substance abuse disorder shared,

They feel that the mom is sick. That’s why mom can’t look after them because their mom is sick. They have no idea where she is. . . Just that mom is sick, and mom can’t look after them. But my granddaughter says that’s okay. We have Nanny.

Only 4 out of the 12 grandparents had permanent custody of their grandchildren. The remaining eight lived with the uncertainty of not having formal custody or guardianship agreements. This caused a tremendous amount of stress for grandparents as they never felt that they had the ability to make decisions on behalf of their grandchild as the legal right to consent remained with their adult child.

Yeah, it’s not quite what I pictured. But anyway, but I mean, he’s lots of fun and like, we love him dearly. But it’s still it is hard. And it’s hard because of all the things that have to be done. It’s not like when you had your own kids, like you made the decisions, but we’re dealing with his parents, too.

The complexities of intergenerational relationships and the dynamics of a grand-family were evident in our interviews. It is important to acknowledge the grief and loss in relationships that can accompany the creation of a grand-family. The change in the relationship between the grandparent and their child living with mental illness and/or a substance abuse disorder was significant. Grandparents spoke about often having to make a difficult choice to prioritize the needs of the grandchild over the needs of their adult child. Many shared that they lived with a constant worry that their adult child would file a report against them, to child protective services, making claims that they were abusive to their grandchild or unfit to be their guardian due to their advanced age. This would then prompt an investigation resulting in the child being re-traumatized and possibly removed from their grandparents’ home and temporarily into foster care. As one grandparent explained,

I have no family other than my son. And right now, he’s really not my family. Because of the meth. Inside him is my son but on the outside, there is box around him and I don’t know who he is. It’s hard to get through that because I’ve been hurt so much. I had to fight my child in court. It was awful to see that he was so sick. I wasn’t able to help him anymore because I had to choose who I wanted to help, [my grandchild], or him. It was awful. So, he was left to struggle on his own.

Participants also spoke of the loss of the role of being a grandparent and the shift that they had to do to become the primary caregiver to their grandchildren. This experience permeated all aspects of their lives. Grandparents often had to step out of the workforce and could no longer engage in their social relationships, hobbies, and travel and enjoy their grandchild in the ways that they had anticipated. Grandparenthood changed and was very blurred. Many grandparents shared in detail how confusing it felt to live in the dual role of grandparent/parent to their grandchildren. “Sometimes I wish we could have just been the grandparents that just were able to spoil him rotten.” Another participant described their perspective:

A normal set of grandparents are the ones that you call up and say, ‘I’ve got scheduled to work tonight. Could you babysit by any chance on Friday and Saturday night?’ Or, the grandparents are calling saying, ‘I’d like to take them for a weekend. Can I take them so I can spoil them rotten and drop them off at your doorstep when I’m all done?’ Fill them up with cotton candy and ice cream and stuff all weekend. There’s none of that. It’s not a normal situation.

### Considering the Future: Connection is Protection

Grandparents often spoke of their future dreams and plans gone awry with the unexpected return to parenting. They voiced the loss of their future; however, in the same breath, the gain of purpose and joy with their grandchildren. Such is the duality of the grand-family experience. Their future has been reshaped by the needs of the child and are greatly focused on the well-being of their grandchild; often coupled with their hope to be alive long enough to set them on a path of well-being.

By stepping up and stepping in to care for their grandchildren, grandparents hoped that they would be a protective buffer so that their grandchildren would not face the same life adversities as their children. They hoped that by forming a grand-family they could mitigate the impacts of trauma that children had experienced in early life. While this was their hope, they too worried about how much damage had already been done to the children due to adverse life events. A grandparent explained,

I just hope that he turns out okay because he’s had a lot of trauma. And I don’t understand it very well, because I don’t know if he remembers it. But they say even if you don’t think they remember it, their brain will remember it. So, I have fear that this trauma will come back. And I hope that it doesn’t, and I hope that he is able to overcome all of the bad stuff he’s experienced and only know the good stuff.

Through sharing their storied experiences in depth during our conversational interviews, grandparents recognized how important they were in the lives of their grandchildren and the positive impact that they were making. They recognized that they were a stabilizing force in the lives of their grandchildren. They realized that the love, safety, structure, and security that they were able to provide to their grandchild could be protective factors that could help break future cycles of trauma and addiction. “Because you want that cycle broken. And you want them to be the best that they can be. But basically, they’re already broken. They come to us already broken.”

Our last interview question focused on the fears and hopes that grandparents had for their future and that of their grand-families. The eight grandparents we spoke with, who did not have legal custodial agreements for their grandchildren, had additional concerns when they thought about their futures related to their own mortality. Grandparents worried about aging and what would happen to the grand-family if they were to die while their grandchild was still under their care. This concern was further heightened with their understanding that without a formal custodial agreement, they did not have decision-making rights in the legal system nor authority to determine future guardianship for their grandchild. As such, they had no right to predetermine who would care for their grandchild and express this legally when planning their wills. For some grandparents, this worry occurred daily. One grandparent shared a sentiment felt by many, “I mean, I’m not old. I’m not young. And I’m scared to die. Because what’s gonna happen to them? Because their parents aren’t mentally able to step up to the plate.”

The dreams grandparents identified for the future of their grandchild were akin to the dreams most parents have for their children. Grandparents wished that their grandchildren would lead happy, healthy, successful lives where their maximum potential could be reached. “I hope that he lives his best life. That he never knows about drugs or alcohol. He doesn’t have to have disappointment.”

Grandparents also spoke about their ultimate hopes for the future of their adult children. These hopes were similar to what they had expressed when speaking about their grandchildren. Grandparents wished that their adult children could also be healthy, happy, and contributing members of society and well enough to reintegrate into their lives again. And at some point, step back into the role of being a full-time parent to their children so that they could become the grandparent that they had imagined prior to needing to lead their grand-families. While this was ultimate long-term hope, most grandparents could not foresee this as ever being their lived realities.

The hope for myself is that I live long enough and to be able to see them through. And that I have enough experience and there’s enough stuff going on that I can lead these kids in the right direction so that they can become viable and stable and that they won’t be hurt by what happened. They won’t end up in this system with mental health system problems because it’s not their fault for what happened and that they will live a good life and that they will be happy, and I hope the same for all the other grandparents who are raising their grandchildren.

The strength of the grand-family is found in the structure, purpose, and direction of their day-to-day life and in planning for the future. Loss and grief are present but are overwhelmed by the love and joy the grandchild brings them. The detailed events of the day are unique to each interviewee and the minutiae of the minutes and hours are tailored to the needs of the grandchild(ren). Grandparents are living in this dichotomy of emotion and feeling. They are full of joy and pride, while also holding grief, loss, trauma, stress, and fear. Their strength is their wholeness as a family and the grandparents’ ability to bear these deep conflicting feelings with uncertainty but grace.

### Supporting Grand-families: What Nurses Need to Know

“It should be easy. You shouldn’t have to fight.” In addition to grappling with their experiences of grief, loss, and trauma, grandparents face countless hurdles and barriers as they navigate legal, education, justice, child protection, and health systems. “You need to push to get things done, and you get lost in the system.”

Compounding feelings of navigation frustrations were feelings of confusion that arose due to conflicting, inconsistent, or a lack of information about supports and services which in turn had a negative impact on grand-family cohesion and well-being. Grandparents spoke of their deep vexations with systems that really did not understand the uniqueness of their family form. This contributed to feelings of shame, retraumatization, and misunderstanding. They could not understand why those in professional capacities were oblivious to their needs and yet did not seek out their input regarding decisions that had a direct impact on their grand-families. “It’s a lived experience. We’re living it, so we have the knowledge.”

Reflecting on these expressed frustrations, we returned to our participants and posed the question: “Grandparents, what do you wish nurses understood better about grand-families?”

Grandparents reflected on this question and shared their responses in writing. Much of the feedback was around feeling unseen and having “to tell them every time I go in that we are a grand-family.” They expressed feeling disrespected and misunderstood by the nurse for the role they have in their grandchild’s life. “Nurses ask, well, are you the parent?” The grandparents felt that nurses have been frustrated with them for not having consent from the parents; however, they are the primary caregiver, and many times the parent is neither reachable nor has the capacity to give consent. “I wish the health care providers would understand we want what’s best for our grandchildren.”

There is a “lack of understanding of the nurse and a lack of system support to recognize grand-families, otherwise, the chart would show it/accommodate it.” A solution suggested by a grandfather was to include in the medical records that the grandchild is adopted or under legal guardianship, so that, “every time you do not have to explain your situation to nurses in front of the children.” This demonstrates their protection of the grandchild, while at the same time reveals their feelings of being unseen and misunderstood, which was “especially true at the beginning of grand-family formation.” These feelings are critical for the nurse to be able to understand the grand-family unit and “not traumatize the child every time.”

## Discussion

### Considering Patient and Family-Centered Care and Family Nursing Care as Approaches to Working With Grand-families

Patient and family-centered care (PFCC) is defined as “an approach to the planning, delivery, and evaluation of health care that is grounded in mutually beneficial partnerships among health care providers, patients, and families.” (Institute of Patient and Family Centered Care [IPFCC], n.d.). PFCC is underpinned by four core concepts: dignity and respect, information sharing, participation, and collaboration (IPFCC, n.d.). These core concepts underpin not only how nurses engage with families for care but also how policies and procedures, education programs, and services are created and implemented to serve the needs of families. In a 2019 scoping review, Kokorelias et al. identified universal and illness-specific aspects of PFCC models of care. The universal concepts identified by Kokorelias et al. include “collaboration and communication; education and support; consideration of the family context; and the need for policies and procedures.” (p. 5).

In addition to PFCC, Family Nursing and the associated theories and principles further support the need to underpin care for families within the context of both health and illness. The International Family Nursing Association (IFNA, 2015) identifies assumptions about family nursing care for health, nursing, and families. These assumptions include health as a dynamic and reciprocal process that affects families collectively and individually; that nurses who work with families are obligated to provide care within the context of the family and to acknowledge the complexity within families and with society, and that families have inherent strengths that will help them navigate health and illness in ways that support the integrity of the family unit. Family nursing is supported by Family Systems Nursing Theory. Family Systems Nursing Theory conceptualizes these assumptions further and identifies families are families are “part of a larger suprasystem and composed of many subsystems; families are greater than the sum of its parts; a change in one family member affects all the family members; and the family is able to create a balance between change and stability” (Shajani & Snell, 2019, p. 26; Wright & Leahey, 2013).

The core concepts and the common aspects of PFCC and Family Nursing models require a definition of family within which to situate these elements. Traditional definitions of a family may create barriers to demonstrating the core concepts of PFCC because families come in many forms. Wright and Bell (2021) define family as “a group of individuals who are bound by strong emotional ties, a sense of belonging, and a passion for being involved in one another’s lives” (p. 61). The simplest definitions of family that best represent the core concepts are “family is who the patient (client) says they are” (Institute for Patient and Family-Centered Care, n.d.; Shajani & Snell, 2019; Wright & Leahey, 2013).

An awareness, acknowledgment, and acceptance of the unique structure of a grand-family by nursing is foundational to understanding the experience of grand-families. The flexibility and acceptance of the definition of family within PFCC and family nursing clears a path for grandparents to actively participate with nursing to partner and collaborate to guide the care of not only children but the entire grand-family. Kokorelias et al. (2019) described education and support and consideration of the family context as universal parts of many PFCC models. Support within PFCC refers to not only support for the care of the patient/client but also the care of the caregiver. The International Family Nursing Association (IFNA) offers a number of Position Statements that highlight the competencies necessary required of nurses who work with families (IFNA, 2013, 2015, 2017, 2018) This universal approach that is underpinned by evidence-based assumptions and competencies can guide care for grand-families that recognizes their unique experience and focuses on the family. Grandparents who are raising grandchildren step into this role and must also navigate multiple challenges related to their parents’ ability to actively parent and provide the necessities of care. These challenges may reflect the impacts of intergenerational trauma and neglect related to the factors such as mental illness, drug, and alcohol addiction.

While this is an intergenerational family issue impacting many sectors and professionals who provide care, it is often approached from a siloed manner whereby practices and policies available for grand-families do not reflect services and supports that are family-centered. This can, in turn, lead to grand-families feeling that their individual and family needs have not been met and may minimize events that are triggering and lead to feelings of judgment and frustration by grandparents as they seek care provided by nurses and other health care providers. As grandparents step up and step in to lead grand-families, they are faced with multiple and complex challenges as they navigate systems and supports designed to address the needs of a nuclear family without consideration of the uniqueness of grand-families or other diverse family forms. Raising awareness for nurses about the complexities facing grand-families can help nurses to provide supportive family-centered care that is responsive and reflective of the uniqueness of grand-families and in turn support all forms of families.

Mitigating the impact of trauma and neglect has been identified as essential for healthy brain development. Early and consistent caregiving by responsive caregivers has been shown to reduce, prevent or even reverse the impact of toxic stress brought about by early trauma and neglect (Center for the Developing Child, Harvard University, 2012). Grand-families need added support for both the child and the grandparent that is ongoing and readily available. Nurses who regularly engage with grand-families in clinical practice settings are uniquely positioned to partner with grandparents and grandchildren to create supportive care environments that not only embrace this unique family structure but also recognize the unique challenges they face and the need to support grand-families to build on their strengths to positively impact the lives of the children in their care. Using a strengths-based, trauma-informed approach to care, nurses could support grand-families in a manner that would promote physical, mental, and emotional health and implement interventions for the family to thrive.

### Opportunities for Nurses to Support Grand-families

Nurses have an opportunity to be an advocate and champion to advance policies and practices that are grand-family inclusive. For example, advocating for changes in health system admission intake forms that include Grand-Families as a family form, could alleviate the stressors caused to grandparents who often must repeatedly explain that they are the child’s guardian and the circumstances that have led to this. Having a place to identify this in the electronic medical record system would present a way for nurses and other health care providers to understand the composition of the family prior to providing care. In addition, maternal child nurses could work with social workers and grandparents with lived experience, to develop grand-family-specific educational materials and a supportive resource directory which highlight services that would be appropriate for and available for grand-families in their local communities.

Nurses work with grand-families across their lifespan. Not only do grandparents step up and step in to care for grandchildren, but nurses are also uniquely positioned in their practice to step up and step in when they care for grand-families. Nurses are working in emergency rooms when a parent is rushed in after experiencing violence or a drug overdose and no one knows what to do with their child who is waiting and crying. Maternal child nurses are present when a baby is born and know before birth that the baby will be immediately placed into the custody of a grandparent. Nurses work in public health and question why the grandparents are there, and the child’s parents have not consented to their immunizations. Hospice nurses provide end-of-life care to grandparents who question them about who is going to look after their grandchildren when they are gone. Each of these experiences creates an opportunity for nurses to provide supportive care that conveys, “I see you and I understand your family.” However too often, grandparents raising grandchildren families do not feel supported or understood. Too often they are repeatedly questioned about their family and why they, as grandparents, are raising their grandchild. This can have unintentional consequences that may negatively impact the mental health of grandparents and grandchildren and trigger past traumatic memories.

## Conclusion

Grand-families are a diverse and unique family form. They are strong, adaptable, and resilient in the face of adversity. When a grandparent steps up and steps in to raise their grandchildren, grandchildren benefit from the love, nurturance, and home-life stability that grandparents provide. Our research provided a forum for grandparents to share their experiences leading grand-families. Working with grandparents, we learned much about the evolution of a grand-family, the many challenges that they have faced due to a societal and systemic lack of understanding about the multiple complexities facing their families, and their hopes for the future. We have also learned about the joy that they experience as they raise their grandchildren.

While much has been learned regarding grand-families in this study, we also acknowledge its limitations. We did not specify gender as a criterion for consideration when recruiting participants. In our study, there was one grandfather. We question if this could be attributed this to the role of women in families who often function in the predominant role of primary caregiver to children. We believe that future research specific to the experiences of grandfathers would be beneficial and provide additional perspectives regarding whether there are gender differences in grand-family caregiving experiences.

This study focused only on the experiences of grandparents; however, we also acknowledge the intergenerational nature of grand-families. We believe that conducting future research exploring the experiences of various members of a grand-family, such as the grandchild and the adult child, could offer a more comprehensive understanding of the lived experiences of multiple family members. Finally, we feel that it would be valuable to conduct a study exploring the experiences of nurses caring for grand-families. This would provide additional perspectives and help illuminate how nurses understand grand-families and care for them in clinical practice.

We believe that our study has the potential to facilitate the development of evidence-based supports and services, that are responsive to the needs, realities, and complexities of grand-families. Through eliciting the stories of experiences of grandparents raising grandchildren, we have uncovered a deeper understanding of not only what makes grand-families unique but also how nurses can effectively work with these families. Framing our study within the context of patient and family-centered care as well as family nursing theory has provided a new perspective to consider when working with grand-families both in clinical practice and in nursing research.
